# Left atrial volume measurements before and after left atrial ablation for the treatment of atrial fibrillation

**DOI:** 10.1186/1532-429X-14-S1-P201

**Published:** 2012-02-01

**Authors:** Jerry D Walker, Sathya Vijayakumar, Eugene Kholmovski, Nathan S Burgon, Alan Morris, Joshua Cates, Christopher McGann, Nassir F Marrouche

**Affiliations:** 1 Cardiology, University of Utah, Salt Lake City, UT, USA; 2 CARMA Center, University of Utah, Salt Lake City, UT, USA; 3 UCAIR, Radiology, University of Utah, Salt Lake City, UT, USA

## Summary

The aim of this study was to evaluate left atrial (LA) volume measurements using cardiac magnetic resonance (CMR) based angiography before and after LA radio frequency (RF) ablation therapy as a treatment strategy for atrial fibrillation (AFIB).

## Background

Recent studies have demonstrated the usefulness of CMR imaging in the management of patients with cardiac arrhythmia. CMR imaging allows for more accurate measurement of various cardiac dimensions. Significant changes in LA volumes have previously been documented in patients who have undergone ablation therapy. However, these studies have relied on simple two dimensional measurement techniques and did not specify how much time post ablation the volumes were measured. More accurate three-dimensional measurement techniques and the serial follow up of LA volume have been lacking and need further investigation.

## Methods

**Figure 1:**
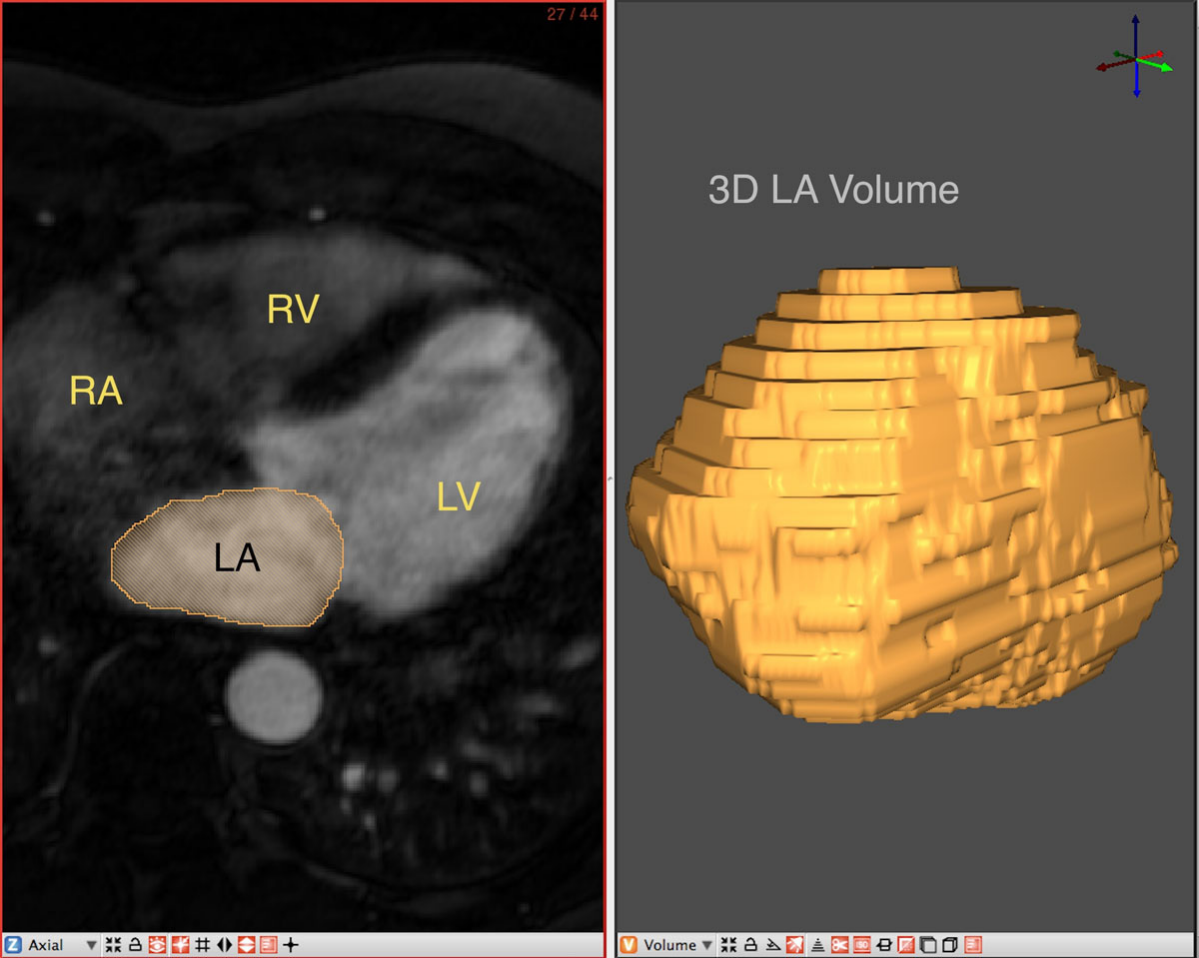
Snapshot of image segmentation and analysis using Corview.

53 patients (45 of whom underwent a single ablation procedure and stayed in sinus rhythm and 8 of whom went back into AFIB and had to undergo repeat ablation procedures) underwent CMR evaluation before and after ablation procedure. Contrast enhanced MR angiography (MRA) with 0.1mmol/kg of Multihance contrast agent (Bracco Diagnostics) was performed in each of these patients on a 3T MR scanner (Verio, Siemens Healthcare, Erlangen, Germany). Typical scan parameters were - axial image volume, TR/TE: 2.8/1.0ms, FOV: 400x400x110, voxel size: 1.25x1.25x2.5mm. Left atrial volumes were accurately calculated using a novel software program (Corview), which allows for segmentation and single slice analysis of individual MRA images as seen in Figure 1. The segmentations were then used to compute the three-dimensional volume of the LA. Data from different acquisitions of MRA at the following time-points were used - Pre-ablation, Immediately Post ablation (<30 hours post), 3 months and 6 months post ablation. The volumes of the LA at various time points were normalized to the volumes measured pre-ablation.

## Results

**Figure 2:**
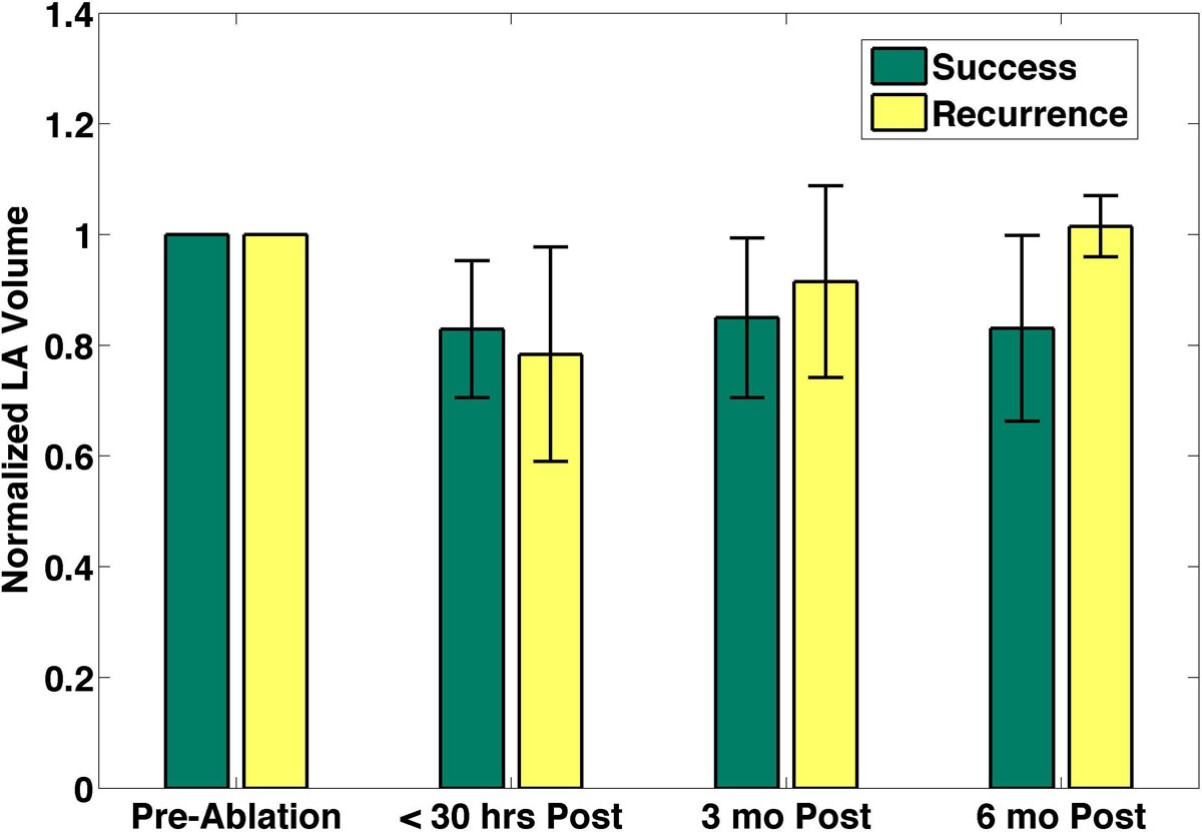
The LA volume measured from MRA normalized to the pre-ablation procedure, of patients with successful outcome and recurrence of AFIB, at multiple time points post ablation.

Accurate LA volumes were estimated using the Corview software program over multiple time points post ablation. It was observed that the LA volume reduced in the patients that were in sinus rhythm after the procedure and stayed the same even 6 months post ablation. However, for the patients that went back into AFIB, the LA volume increased to higher than the pre-ablation value by the time 6 months had passed since the ablation procedure, as seen in Figure 2.

## Conclusions

CMR imaging in conjunction with newer software capabilities can more accurately measure cardiac chamber volumes. It is interesting to note that the LA volume as a function of time post ablation procedure may be used as a predictor for procedure outcome.

## Funding

None.

